# Ethics and applications of isotope analysis in archaeology

**DOI:** 10.1002/ajpa.24992

**Published:** 2024-07-01

**Authors:** Chris Stantis, Benjamin J. Schaefer, Maria Ana Correia, Aleksa K. Alaica, Damien Huffer, Esther Plomp, Marina Di Giusto, Blessing Chidimuro, Alice K. Rose, Ayushi Nayak, Ellen J. Kendall

**Affiliations:** ^1^ Department of Geology and Geophysics University of Utah Salt Lake City Utah USA; ^2^ Department of Anthropology, Gender and Women's Studies, and Latin American and Latino Studies University of Illinois at Chicago Chicago Illinois USA; ^3^ The Center for the Recovery and Identification of the Missing University of Illinois at Chicago Chicago Illinois USA; ^4^ Division of Anthropology The Field Museum of Natural History Chicago Illinois USA; ^5^ Interdisciplinary Center for Archaeology and the Evolution of Human Behaviour (ICArEHB) Universidade do Algarve Algarve Portugal; ^6^ Laboratório de Arqueologia e Antropologia Ambiental e Evolutiva Universidade de São Paulo Sao Paulo Brazil; ^7^ Department of Anthropology The University of British Columbia Vancouver British Columbia Canada; ^8^ Department of History Carleton University Ottawa Ontario Canada; ^9^ School of Social Sciences University of Queensland Saint Lucia Queensland Australia; ^10^ The Alliance to Counter Crime Online Washington DC USA; ^11^ Faculty of Applied Sciences Delft University of Technology Delft The Netherlands; ^12^ Museu de Arqueologia e Etnologia, Universidade de São Paulo Sao Paulo Brazil; ^13^ Department of Geography and Environmental Science University of Reading Reading UK; ^14^ Department of Archaeology Durham University Durham UK; ^15^ Department of Archaeology Max Planck Institute of Geoanthropology Jena Germany

**Keywords:** accessibility, archeological science, equity, research ethics

## Abstract

This synthesis explores specific ethical questions that commonly arise in isotopic analysis. For more than four decades, isotope analysis has been employed in archeological studies to explore past human and animal dietary habits, mobility patterns, and the environment in which a human or animal inhabited during life. These analyses require consideration of ethical issues. While theoretical concepts are discussed, we focus on practical aspects: working with descendant communities and other rights holders, choosing methods, creating and sharing data, and working mindfully within academia. These layers of respect and care should surround our science. This paper is relevant for specialists in isotope analysis as well as those incorporating these methods into larger projects. By covering the whole of the research process, from design to output management, we appeal broadly to archaeology and provide actionable solutions that build on the discussions in the general field.

## 1 INTRODUCTION

Isotope analysis is increasingly a key part of the archeological toolkit in today's method and practice, approaching the commonplace in multidisciplinary archeological studies (Roberts, 2022). This suite of tools can provide information about a breadth of topics such as diet, climate, migration, and health, with a resolution capable of inferring change over 1000 of years across broad cultural groups or small slices of months within a single person's lifespan. Modern plant, animal, and human samples contribute to mapping the geological, ecological, and metabolic processes that shape isotopic values. When these isotopic values are shared via large‐scale open databases it allows researchers to compare data easily, bolstering statistical power, and making connections beyond the scope of original research questions.

**FIGURE 1:**
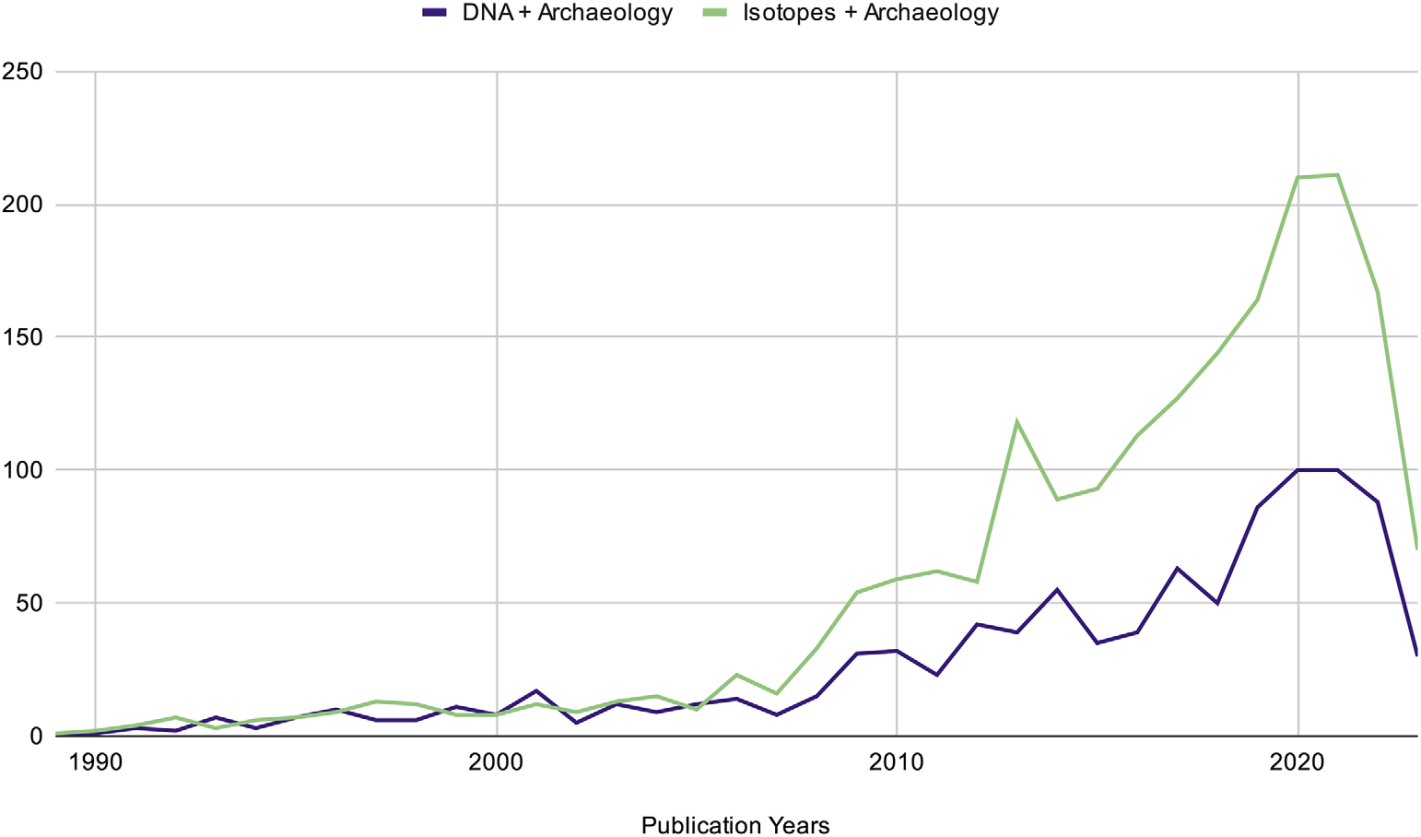
Web of Science search results for two terms, “DNA Archaeology” and “Isotopes Archaeology,” by year.

Within the ever‐maturing field of isotopic analysis, application of techniques increasingly raises important ethical issues. Ethical practice has become a shared focus across archaeology, a discipline which, at its core, destroys irreplaceable context in order to construct knowledge (Crellin & Harris, 2020; González‐Ruibal, 2018; Squires et al., 2019, 2022; Turner et al., 2018). In the last 5 years, archaeology has seen an increase in academic discussion regarding the ethical use of ancient DNA (aDNA) (Argüelles et al., 2022; Austin et al., 2019; Booth, 2019; Fleskes et al., 2022; Fox & Hawks, 2019). In many respects, the history and development of isotope analysis mirror the trajectory of aDNA: increasingly routine use across subdisciplines of biological and forensic anthropology, inherently destructive sampling methods, and arising issues of “big data.” While extraction and analysis of human tissues are common to both aDNA and isotope analyses, the ethics of isotopic analysis should be considered in light of the specific issues and implications raised by biogeochemical analysis. Furthermore, the wide reach and accessibility of isotopic research to nonspecialist audiences obligates researchers incorporating isotopic analyses to consider the wider impacts of their work, and to promote improvements in ethical standards in biomolecular archaeology in keeping with responsible research. In this manuscript, we highlight how growth in the application of isotope analysis has increased the need for specific ethical guidelines and raised concerns specific to the discipline (see Figure 1).

This paper begins with a broad question: what are the levels of care and respect we should integrate into our research design and practice as isotope specialists? We propose some isotope‐specific solutions within this paper, including methodological approaches to mitigate destructive analysis of invaluable human remains and specific examples of open data access and public engagement at the forefront of isotopic research in archaeology. Some problems are unique to isotope analysis; one such example is the issue of destructive volume. Decreasing analytical costs have driven mass application of isotope analyses across the world, and while there are increasingly smaller sample volumes for certain suites of isotopic analyses, such as microsampling of dentine (e.g., Curtis et al., 2022; Czermak et al., 2020), this gain has been offset by increasing permutations in the range of complementary isotopic analyses. Some topics of particular concern to isotopic analysis also have broader applicability to other subdisciplines. For instance, we address the responsibility of researchers approached by collectors, dealers or auction houses to “test” remains and artifacts, an increasingly likely scenario. We also discuss the responsibilities of members of the academic community to each other, to form a supportive community that encourages a diversity of perspectives, which enrich the discipline. By covering the whole of the research process, from design to output management, we appeal broadly to archeology and provide actionable solutions. Furthermore, we target audiences concerned with communities and methodological issues, which are not always well integrated into a synthetic approach to ethics. In summary, there is a growing need for discussion and codification of ethical considerations in planning research that involves isotope analysis. Here we present a holistic approach to addressing the unique range of ethical concerns in isotopic studies.

So far, various scholars have articulated guidelines for reporting and visualizing isotope results (Roberts et al., 2018; Stantis, 2021; Szpak et al., 2017; Vaiglova et al., 2022), but here we address, in fairly plain language, the gap in ethical issues that surround isotope analysis in archaeology. Namely, beyond “to sample or not to sample?,” we consider a range of questions around the ethics of who, how, and when to sample, the impact of data beyond research projects, and issues of access and inclusion within research. To do this, we organize the paper to touch on our principal obligations for ethical work: working with descendant communities and other ethical rights holders (defined here as those who have ethical entitlements or enforceable claims in relation to decisions surrounding cultural heritage), choosing methods, creating and sharing data, and working mindfully within the academic community. These many layers of respect and care for individuals and communities should surround our science. We wrote this paper to be of use for those specializing in isotope analysis as well as those incorporating these methods into larger projects.

### 1.1 Positionality

It is known that positionality influences research (El Kotni et al., 2020; Foote & Gau Bartell, 2011; Moffat, 2016). Positionality statements in manuscripts help researchers reflect on their worldview and assumptions as well as help readers in understanding what influences may be between and behind the lines. There is no succinct way to explain 11 authors' positionalities and “reflexivity is not a panacea” (Darwin Holmes, 2020, p. 4) for understanding researchers' motives and positions, but we provide some brief overview of our composition and values.

Many of us have not previously collaborated, and have yet to meet in person. All of us consider ourselves scientists who use or have used isotope data to answer questions about the past, and we all want the continued creation of this data to address archeological questions. At the time of publication, the majority of us work in academia without the benefit of long‐term job security, occupying temporary positions (sometimes multiple concurrent positions), or positions not associated directly with archeological projects. Additionally, some of us are disabled, neurodivergent, and/or have caring responsibilities alongside work. Some of us are graduate students. Some of us are first generation academics. Many of us are white, raised in the Global North, or are affiliated with institutions in the Global North, while others had their entire educational and academic pathway in the Global South and without English as their mother tongue. Many of us have had the privilege to work with Indigenous ancestors and communities, yet none of us identify as Indigenous.

This paper was written in a collectivist manner. The first author organized meetings, facilitated group discussions, provided suggestions for group work and structure, and wrote the introduction and conclusion. The main sections were written by subgroups, and all contributed to editing.

## 2 RESPONSIBILITIES TO DESCENDANT COMMUNITIES

Researcher collaboration with descendant communities and the general public needs to be reconsidered to develop not only an ethical but also a complete isotopic bioarcheology at the community level. The requirements of descendant communities must be given first priority in isotopic research, ahead of scientific inquiry and knowledge formation. Far from hindering scientific inquiry, mutually consensual, mutually beneficial collaboration between scientists, and descendant communities can be enriching, producing better science with more impactful outcomes. A recent example of such an approach is a partnership between Coast Salish communities in the Pacific Northwest of North America and scientists, integrating Indigenous knowledge and science (including isotopic analysis) to share and generate improved understanding of the lives of woolly dogs in Coast Salish history (Lin et al., 2023). Unfortunately, such partnerships are not the norm, and legal protection against exploitation may fall short, highlighting the urgent need for within‐discipline ethical expectations for engaging with descendant communities. Human remains are often not protected by national legislation intended to safeguard them (Stantis et al., 2023), even though many countries have passed laws meant to do so, such as the Native American Graves and Repatriation Act of 1990 (NAGPRA, Pub.L. 101–601; 25 U.S.C. 3001–3013;104 Stat. 3048–3058). However, these laws often do not protect all ancestors. For example, in the United States, there is an increasing call to protect the graves of African Americans, which currently have no legal protections (Dunnavant et al., 2021). Abuses of existing power differentials will persist unless there is legislation that ensures actual penalties for those who disrespect the dead, whether that be through unethical extractive procedures or authenticating the market for antiquities or human remains (Graham et al., 2021). Moreover, researchers must hold themselves to high ethical standards that exist independently of extant legislation.

The isotope bioarcheology research community must prioritize ethical research design and practices, even though scientists often lack the institutional support for people‐focused ethical techniques, often best aligned with a “slow science” approach, which fosters communication between knowledge sharers. A plea has been issued emphasizing that the engagement of rights holders remains an important stage in the advancement of archeological science (Pilaar Birch & Szpak, 2022). This aspect is crucial in archeological work, but is often overlooked in research designs that apply isotopic methods to archeological, bioarcheological or forensic questions. When research objectives are refocused to align with the needs of communities or are community‐led from the onset, there are more opportunities for positive community outcomes (Flexner, 2021).

It is also important to guard against promoting false dichotomies of scientists on the one hand, and the descendant communities whose ancestors have been routinely studied in extractive research on the other; dual identities are intertwined for many researchers (Aranui, 2020; Fox, 2020; Lippert, 2006; Supernant, 2018; Tsosie et al., 2021). We instead argue that the notion of extractive research conducted by outsiders in opposition to a resistant group of rights holders is a tired trope, benefitting neither side. Rather than focusing on the status quo, we posit that ethical research design disrupts and remediates neo‐colonial institutional abuses.

### 2.1 Collaboration and consultation with descendant communities and other rights holders

By prioritizing the needs of descendant communities, archeology fosters a multivocal dialogue that interrelates the traditional and scientific spheres of knowledge. Communities need to be recognized as actors that contribute to the construction of archeological narrative (Colwell, 2012). This approach has been widely used in archeological research in different parts of the world that may serve as guiding examples, many outside the Global North (Figueiro, 2024; Gblerkpor & Nkumbaan, 2014; McKinnon et al., 2014; Silva et al., 2011). Isotopic bioarcheological research could therefore become even more global and diverse in practice, fostering a multivocal approach by placing the needs of descendant communities alongside scientific research and prioritizing those needs above scientific inquiry. This approach becomes even more important when studying human remains, since, for many communities, these represent much more than a scientific resource. Embracing this approach not only enriches the discipline but also fosters a more inclusive and respectful engagement with the past.

In this modality, knowledge is co‐produced between archeologists and explicitly acknowledged local collaborators and communities. For instance, local collaborators often have a deep knowledge regarding their own geography, history, and the local distribution of archeological sites (see Rocha et al. (2014), who report on collaborative work with traditional communities in the Brazilian Amazon), especially in comparison to foreign researchers who only visit briefly for sampling without the involvement of descendant communities. Thus, these collaborations provide mutually‐beneficial, important insights into the interpretation of the archeological data generated. However, achieving these collaborations is not without their challenges.

In South America, we can cite two examples where local researchers have been included in archeological projects. In Peru, the Ministry of Culture (previously Instituto Nacional de Cultura) requires all archeological expeditions to have a Peruvian co‐director, who has been approved by the ministry. However, while well‐intentioned, this can relegate Peruvian archeologists to a subservient or token role and reduce their expertise to just a signature. In Brazil, universities located outside capital cities have historically been overlooked in favor of capital‐based universities (Paladino, 2012), which may create challenges, for instance, for Indigenous descendants to engage in research projects. Involving local university students, such as those from Indigenous communities from the Amazon region, within research projects contributes to their long‐term involvement and recognition in scientific research, thereby dismantling the center–periphery relationship in the production of knowledge (Argüelles et al., 2022).

Centering community engagement and collaboration provides a space for more robust scientific inquiry, though it must be expected that community needs may not always align with researchers' sense of urgency for co‐produced output (Mason, 2021). The pace of scientific research tends to move rapidly, especially in isotopic research where methodological improvements allow data to be obtained within weeks and submitted for publication shortly thereafter. While traditional publication of isotopic data in journals and books might be experiencing the same lag effect as all other fields due to reviewer shortages, the emergence of preprint repositories such as BioRxiv offers a potential solution. However, lessons from slow science (Stengers, 2016) push us to realize that rapid science is not always good science. The slow science movement, developed as a critique of the “publish or perish” mentality and output‐centered performance evaluations, pushes for methodical, steady research, with a more engaged, critical, and humane academic workload (Cunningham & MacEachern, 2016). At its core is the idea that studies need the time and space to build on each other incrementally, and that published research that represents small steps in a process, or the clarification of methodological or ethical issues, will still count as valid research and contribute to scholar metrics. This movement toward slow science also provides time to think about a central question in all of our research: is isotope analysis always beneficial or appropriate?

For effective community engagement in research, the “three Ps” have been suggested: “patience, politeness, and persistence” (Poor & Woods, 2022). For example, decades of collaboration between the Huron–Wendat First Nation and archeologists/isotope specialists resulted in a rigorous and community‐centered research program (Forrest et al., 2020). Ancestral Huron–Wendat remains in Central Canada were linked to descendant communities through oral histories and community affiliations. This example is essential to our argument that ethical work and care are possible in isotope research through community‐centered project design. Ongoing collaborations with Huron–Wendat descendants allowed for consensus to be built around, which human tissues would be analyzed, with teeth being considered the main skeletal element that was suitable by both communities and researchers. In this case, focusing on dental analysis to elucidate dietary trends among Huron–Wendat ancestors not only advanced scientific goals but also fostered a balanced approach to human remains, respecting these rights holders' perspectives. It provided a powerful set of samples to address numerous questions about human life histories.

Finally, ethical archeology must prevail in the perspective of collaborating with law enforcement (if we posit that law enforcement officers are also rights holders in certain scenarios). Law and science follow different methods or processes: science intends to be self‐correcting whereas law enforcement action and case prosecution are often time‐sensitive. Thus, for effective and ethical collaboration, isotope bioarcheologists need to understand how evidence is used in the judicial system and the judicial system needs to understand how stable isotopes may help in the analysis of physical evidence (Cerling et al., 2016). In the field of forensic anthropology, multi‐isotope profiles combined with isotopic landscapes have contributed to human identification, providing evidence on birth region, adult residence, recent travel, and dietary choices (Bartelink & Chesson, 2019). One of the most well‐known examples is that of “Saltair Sally.” In 2000, the remains of a young woman were found in Utah, and despite extensive search efforts, the murder victim was not initially identified. In 2007, oxygen analyses of the victim's hair allowed researchers to reconstruct her movements in the 22 months prior to death, based on the principle that oxygen isotopes values vary in local water and that this signature is incorporated into hair as it grows. With this new information, the police were able to better target search efforts and in 2012 identified the victim through DNA analyses (Cerling et al., 2016).

Consideration should also be given to how descendant communities may become victims of heritage or environmental crime, and the role that may be played by isotope researchers in increasing or decreasing harm. For example, in the course of investigation and prosecution law enforcement may be concerned with reproducing the full chain of custody from source site/country to market, to one or more private collectors or collecting institutions, and often to market again. Evidence may emerge that a person of interest, such as a private collector, dealer, or representative of an auction house, gallery, or ethically questionable institution approached specialist laboratories seeking authenticating analyses. These laboratories may play a key role in reducing or perpetuating harms that result from crime, based on their response. Adequate preparation for the realities of today's global antiquities and human remains trade arguably requires the development of a code of ethics that commercial laboratories can adhere to if they are approached by private individuals. Such individuals may not always be transparent about their role as collectors or dealers, their intentions to sell, or their need for additional archeological context to increase the realized price. This suggestion of a code of ethics for stable isotope laboratories is not novel but is inspired by similar initiatives in aDNA and radiocarbon research (Ávila‐Arcos et al., 2022; Cortez et al., 2021; Hajdas et al., 2022). The use of stable isotope analyses in forensic settings is still not widespread, partially due to a lack of international and validated standard operating procedures, but its use is steadily increasing (Bartelink & Chesson, 2019).

Researchers embarking on isotopic analysis should be ready to reprioritize projects if it becomes clear that the ethical conditions to carry out destructive sampling are not met. In summary, researchers working with partners at different points of the knowledge‐power gradient should aim to establish open communication that develops capacity outside the main centers of knowledge production. This can be achieved through some activities, including training and mentoring local researchers, active participation in collection curation and analyses, and providing financial support for personnel, equipment, or technological infrastructure (McKerracher & Núñez‐de la Mora, 2022). These contributions will, of course, vary depending on the size of the project and the career level of the researcher.

## 3 DESIGNING ETHICAL METHODOLOGIES FOR DESTRUCTIVE SAMPLING

When designing an archeological project, the choice of methods should be carefully and continuously evaluated before, during, and after sampling. This is especially important when considering destructive analyses such as an isotopic approach since archeological material is finite and destructive analyses impact future research (Mays et al., 2013; Pálsdóttir et al., 2019). Our ethical stance is that destructive analyses should be kept to a minimum and that researchers should have a clear hypothesis or question at the onset of the project (Baker & Worley, 2019). We also echo Squires et al.'s (2022) call for the integration of ethics committee reviews before research involving human remains begins, whether they include destructive analyses or not, to advise and promote policies and procedures that reflect our current ethical standards as a discipline.

### 3.1 Consider complementary analyses and futureproofing

Besides isotopic analysis, there are many other analyses that can be carried out on biological material and new avenues of research are constantly being developed. Potential complementary analyses (though also destructive to varying degrees) include radiocarbon dating (Sánchez‐Cañadillas et al., 2021), sex estimations through enamel peptide etching (Parker et al., 2019; Stewart et al., 2016, 2017), ancient human, faunal and pathogen DNA (Orlando et al., 2021; Spyrou et al., 2019), steroid hormones (Quade et al., 2021), and zooarcheological identification by mass spectrometry (ZooMS) (Buckley et al., 2009). A myriad of complementary analyses can also be carried out on dental calculus (Chidimuro et al., 2022; Radini & Nikita, 2022). Researchers should consider how the application of multiple methods may intersect with isotopic analyses on a practical and interpretative level. In particular, the application of some of these methods may help inform isotopic project design or may even reduce the need for destructive isotopic analyses (Chidimuro et al., 2022). For example, using minimally destructive ZooMS methods, McGrath et al. (2019) determined that bear *and* human bone were worked into the Iroquoian bone points from the Droulers, McDonald, and Mailhot‐Curran assemblages *c*. mid‐14th to late 16th centuries CE in southern Quebec, Canada. This not only advanced the way that mixed methods can be used to identify the presence of ancestral remains but also how isotope investigations can then be subsequently planned with descendants and other rights holders to avoid continued handling of ancestral remains.

Because of the research potential of biological material, a logical, sequential sampling procedure should be planned ahead of destructive sampling to avoid limiting information that can be obtained. The order of analyses should be carefully considered and communication and cooperation between multiple specialists may be necessary to determine the best sequence. To obtain the maximum amount of information and to ensure that certain protocols do not negatively affect one another, methodological modifications may need to occur such as wearing extra PPE to avoid unnecessary contamination. Even if multiple analyses are not currently planned, efforts should be made to consider potential future research (such as systematically recording and collecting dental calculus when preparing teeth for isotope analysis). Involved researchers should be sufficiently competent and experienced to conduct the work proposed, which includes understanding if enough isotopic variation is present in the specific archeological context to adequately address the posed question (Mays et al., 2013) or any method particularities, such as sample pretreatment, that may affect biological signatures or the applicability of other approaches (Pellegrini & Snoeck, 2016; Snoeck & Pellegrini, 2015).

### 3.2 Not all samples are ethically equal

There are many types of human and animal‐derived materials that can be analyzed isotopically, including (but not limited to) bone, tooth dentine and enamel (Sealy et al., 1995), hair (Mora, 2022), wool/textiles (Von Holstein et al., 2016), leather (Spangenberg et al., 2010), parchment (Doherty et al., 2023), ivory (Coutu et al., 2016), shell (Leng & Lewis, 2016), feathers (Capriles et al., 2021), and coprolites (Witt et al., 2021). Each material incurs differing ethical considerations and certain tissues may hold different degrees of importance in different cultures.

Globally, regulation and attitudes to the destructive sampling of human biological tissues vary widely, and so destructive sampling should always be considered within the legal and cultural framework specific to the material (Márquez‐Grant & Fibiger, 2011). Some countries have clear legal and/or ethical distinctions between forensic, archeological and paleontological biological material, while others do not and legislation specifics vary widely (Hutt & Riddle, 2007; Márquez‐Grant & Fibiger, 2011). In the United States, NAGPRA is a legislative framework that guides the excavation, analysis and repatriation of human remains for Indigenous nations. However, in other regions, beyond permissions that must be obtained from government bodies, research collaborations may not even inform descendant communities or other rights holders of destructive work. In many South American countries such as Argentina, Brazil, Chile, Peru, and Uruguay, regulatory bodies and protectionist laws were created in the 20th century to regulate archeological sites, including the excavation and conservation of human remains. These regulations may have protections regarding the export of human remains to foreign countries, but repatriations of said remains to Indigenous groups—like NAGPRA, the Aboriginal Heritage Act in Australia, the Maori Repatriation Act in New Zealand, and the Indigenous Cultural Heritage Act in Canada—are few and far between (Ponce, 2011). Indigenous groups in this region sometimes are not aware of the existence of these collections, and their connection to them has been broken through colonial practice, a practice continued and reinforced during the dictatorships that took hold of the continent in the mid‐20th century (dos Santos, 2019). Restitution efforts are often hindered by opposition from human rights and heritage legislation (Cury, 2020; Verdesio, 2011), even though initiatives are underway by some researchers/projects to contradict these practices (Cury & Bombonato, 2022; dos Santos, 2019). It is clear that an entrenched national (or even global) ethical protocol must be developed that works toward meeting the standards of innovative projects in our field.

Generally, destructive analysis of faunal material is not subject to the same kind of ethical considerations that human material may be and is often less rigorously controlled (Pálsdóttir et al., 2019). In some instances, using fauna as proxies may help minimize destructive analysis of human material. For example, the mutualistic and symbiotic relationship that exist between humans and dogs is a powerful way to trace human diet and mobility. Guiry (2012), Guiry and Grimes (2013) propose the framework of the canine surrogacy approach that focuses isotopic work on dogs to shed light on human subsistence patterns. This has been used in the context of collaborative research with Huron–Wendat ancestral remains, where Glencross et al. (2020) consider the dietary variation in “old dog bones” to interpret the types of resources that ancestors were consuming at Tay Point. These multispecies proxies to human food sourcing are not limited to dogs, but also other commensal agents such as rodents with caveats, (see Guiry and Gaulton (2016)). However, the increase in destructive analysis of archeofaunal remains has raised ethical issues and sampling of these remains should be undertaken with the understanding that they are also a finite resource (Pálsdóttir et al., 2019). We must also remember that some communities might view other‐than‐human animals as part of their extended kinship system or of some other particular significance (Kohn, 2007; Legge & Robinson, 2017), and that anthropogenic modification of animal‐derived material, such as the use of feathers in culturally significant garments, may alter the significance of the material and thus raise ethical considerations. For example, the Wari feather panels from Churunga Valley, Peru are composed of 1000 of macaw feathers and intricately woven into finely crafted textiles. These panels were found in a large cache of ceramic vessels and likely relate to important burial practices of the era (King, 2013). While isotope analyses of these feathers could contribute to understanding the trade and management of macaws (Capriles et al., 2021), removing feathers for isotope analyses can introduce other ethical concerns relevant to maintaining these artifacts in their entirety not only for curation but also symbolic integrity (Alaica et al., 2022).

### 3.3 Determine what and how much to sample

In an archeological context, it is likely that the biological material recovered is limited by factors such as site type, excavation size, preservation, and fragmentation. This will impact the isotopic analyses that can be undertaken and it should be taken into consideration whether the material available is suitable for the research questions posed. Care should be taken to avoid key anatomical landmarks, pathologies, and anthropogenic modifications. The location to be sampled (for example, sampling enamel from the lingual surface instead of the buccal surface of a selected tooth) is also crucial to consider, in terms of the impact on the appearance and integrity of human remains following sampling. Sample sizes required to answer these questions should be considered on multiple scales: the number of individuals, the number of tissue samples, but also the physical amount of tissue needed. If collaborating with an isotope laboratory, researchers should check the laboratory website or contact them directly to confirm minimum sample size by weight. This process may need to be reflexive, for example, if the recommended sample size for carbon and nitrogen isotope analysis is routinely producing much more collagen than needed for the measurements required, then the amount of material sampled should be reduced. While there is a temptation in archeological research to sample as much as possible within material and budget constraints, a researcher should consider what is the minimum number of individuals or tissue samples needed to answer questions satisfactorily.

Recommendations include employing statistical techniques such as power analysis or conducting pilot studies that may help determine optimal sample sizes (Vaiglova et al., 2022). Researchers can consider planning the study more formally in the form of a preregistration, a document in which the research design, and sometimes hypotheses is specified before research is carried out (Ross & Ballsun‐Stanton, 2021). In some museums and curating institutions, this is becoming required documentation, and so including this in a research group's routine plan may streamline permissions and access. The aim should be to collect the minimum amount of tissue possible for the destructive analysis planned. As methodological techniques develop, the amount of material required for each analysis is generally decreasing; however, the suite of potential analyses is increasing, resulting in continued demand for finite resources.

### 3.4 Assess sample quality

Ideally, prior to destructive analyses, determine whether the samples selected are likely to produce isotopic data to reduce unnecessary destruction of material. Several advanced techniques exist to address collagen preservation and potential diagenesis, such as micro‐computed tomography (micro‐CT), laser‐induced breakdown spectroscopy (LIBS), Fourier Transform Infrared (FTIR) spectroscopy, and Raman spectroscopy (Rusak et al., 2011; Tripp et al., 2018). For instance, bones with high cortical porosity calculated from micro‐CT are unlikely to contain collagen, but this approach is more powerful when combined with some form of spectrometry that informs on chemical composition (Tripp et al., 2018). LIBS is minimally destructive since it gradually laser‐ablates the bone surface to measure atomic ratios of the resulting vapor, wherein lower Ca/F ratios indicate lower collagen preservation (Rusak et al., 2011). FTIR analyses assess the extent of bone diagenesis before stable isotope analysis of bone carbonate to ensure the exclusion of the most diagenetically altered samples (Kontopoulos et al., 2018). Raman spectroscopy offers a nondestructive screening technique capable of identifying alterations to the composition and integrity of collagen in bone (France et al., 2014). Application of these types of techniques may not always be practicable or necessary, but they are especially pertinent for particularly ancient and/or scarce material. In other cases, at the very least, researchers should draw upon or conduct isotopic pilot studies so that the suitability of a small quantity of material can be tested before larger‐scale destructive analyses.

### 3.5 Preserve through recording

All samples, as well as the sampling and analytical process, should be properly documented for posterity. To prevent the potential loss of vital information, it should be ensured that all material has undergone appropriate macroscopic analysis in accordance with accepted standards. For example, human remains should be analyzed by a trained osteologist following internationally recognized osteological recording methods (e.g., Buikstra & Ubelaker, 1994; Mitchell & Brickley, 2017). Detailed zooarcheological and paleoethnobotanical analyses must be conducted on any nonhuman species proxies before isotope analyses. This type of documentation may already exist. However, in some cases, the information may need updating or it may even be necessary to undertake new analysis in association with appropriate specialists; authors in this group have encountered collections where sex, age, or ancestry estimation were determined using outdated techniques, and/or there were unrecorded pathologies. Imaging technology that can preserve morphological information should be employed to safeguard the material. At the bare minimum, samples should be adequately photographed, both before and after sampling, representing both the sample and its source. Photogrammetric models can be built from images taken using a mobile phone, although the quality achieved is more adequate for visualization and educational purposes than qualitative and quantitative studies (Edelmers et al., 2022; Li et al., 2020). CT‐scanning preserves both internal and external morphological information and aids in diagenetic evaluation. Although access to CT‐scanning is expensive, it may be possible to establish collaborations with local medical and veterinary hospitals, particularly those within universities. Some of these institutions charge a fee (although one of the authors' experience shows that this is more true of the Global North than the Global South, where collaborations can be established in exchange for co‐authorship or simply mutual support, especially when dealing with small samples such as teeth or bone fragments, which can be CT‐scanned rapidly). Moreover, we encourage students and researchers to enquire about these possibilities locally, while also urging funding bodies to allow resources to be funneled toward these ethical practices. Incidentally, such imaging techniques also contribute to the curation and management of cultural heritage (Weber, 2015), and thus create beneficial information in itself. For teeth, dental molds may also be taken. These reproduce overall morphology, but also macro and microwear, which may be used to infer diet and other masticatory behaviors (Scott & Halcrow, 2017; Ungar et al., 2008). Molding materials are relatively accessible and simple protocols exist to apply them to archeological material and, depending on the material used, these molds maintain resolution for several years (Fiorenza et al., 2009; Galbany et al., 2006; Sawaura et al., 2022; Scott et al., 2006).

### 3.6 Plan what to do post‐sampling

An ethical destructive sampling methodology should have a robust post‐sampling plan. Designed in consultation with relevant institutions, communities and rights holders, this plan should include the dissemination of data and findings as well as what to do with any remaining materials. Access to physical samples, much like accessing the data and publications generated from them, is fundamental to reproducibility and transparency in archeological science. Additionally, unequal access (to both data and opportunities to participate in production of data) reinforces global power imbalances and can negatively impact colleague's careers, communities' interests and trust, and the future of research as a whole (Cinnamon, 2019; Graves et al., 2022). Isotopic researchers should aim to ensure equal access to material and data. For example, researchers should plan to return any material remaining after analyses to the community or institution of origin. There is the caveat that not all institutions necessarily want or have the facilities to store remaining material returned—other options could be explored, such as an open access database of “orphaned” material available for further analyses while stored with the laboratory. If further analysis is required, a new application must be evaluated, justifying the reasons for access renewal. All data produced (including any new macroscopic analysis) and short reports of the work done should be shared with rights holders, and to the broader society where possible. With these invested groups having potentially diverse backgrounds and cultures, consider culturally sensitive means of sharing findings (Ross‐Hellauer et al., 2020).

## 4 ETHICS OF DATA MANAGEMENT AND STORAGE

Once isotopic data is collected, many ethical implications are still present in its curation and future use. One of the possible paths to mitigate the impact of the destructive nature of isotopic analyses lies in pooling and reusing collected data to answer novel research questions (Plomp et al., 2022). Sharing research data diminishes the gap between researchers, institutions, and funding bodies that are able to afford extensive and/or expensive data collection and those who are not. However, researchers and communities face many challenges in how to share data in ways that are sustainable and equitable, given the multiple rights holders involved in the production of said data, the long‐term costs of maintaining data (both in terms of money and labor) and the fact that data access is not equitably distributed (Fleskes et al., 2022; Goñalons et al., 2023). It is also essential that planning around storage of post‐processed samples is considered before the project begins. Undertaking isotope analyses does not always exhaust the collagen, enamel, or other tissues extracted in these procedures. Therefore, it is essential that planning begins early on about how these samples are stored and curated. To tackle these issues on data management, we outline in this section the importance of data management plans, the principles that should guide data sharing with ethical rights holders, the management of sustained community engagement, and provide some examples of data repositories. We reiterate here the importance of “slow science” outlined in other sections of this work, as we believe data should survive beyond a researcher's working life (Argüelles et al., 2022; Cunningham & MacEachern, 2016). Hence, we encourage funding bodies and institutions to support and demand research that considers implications beyond the life course of the projects (McKerracher & Núñez‐de la Mora, 2022).

### 4.1 Data management plan

Part of the ethical treatment of isotopic data is carefully planning data collection, treatment, and management. A tool to facilitate this planning is the Data Management Plan (DMP, or Output Management Plan). These types of plans are now often required by funding agencies (NIH, European Commission, NWO, and others). We argue that whether your institution or funder requires you to set up a plan or not—you can use it to ensure that data will be managed and shared responsibly. Generally, the templates for these plans contain guiding questions that allow for consideration and reflection around data responsibilities, such as those provided by the Turing Way community (Data Management Plan—The Turing Way, n.d.). These plans guide the processes for planning data storage, minimizing the risks of data loss, and outlining the process of data documentation. Many templates also consider the costs of data management, whether this involves the labor involved in data management itself or the costs of long term preservation. By using these DMPs you can consider from the onset of a project whether the data can be shared openly or not. IsoArcH team members have released a DMP that aligns with future upload of bioarcheological data into IsoArcH, although it could be used for any project planning to generate isotopic data from bioarchaeological research (Plomp et al., 2024).

### 4.2 OpenData

Where possible, the isotopic data should be made openly available, although exceptions exist as discussed below. Open data can be shared and reused with minimal restrictions, allowing for more sustainable use of the data and maximizing the research impact and visibility the data can have. By sharing data more openly, we increase the transparency of the research process. This transparency facilitates validation of interpretations, helps discover mistakes and thus ingrains confidence that the research was conducted following ethical principles and methods (Marwick et al., 2017). If interpretations and conclusions can be validated, isotopic research will become more reproducible (see Karoune & Plomp, 2022). Open data has the potential to make research more equitable, as access to research data is no longer based on academic positions or institutes. Open data can also facilitate collaboration, and bring in a wider range of perspectives if participation of other rights holders (whether individual citizens or communities as a collective) is encouraged. Open data can also prevent expensive re‐collection of data, and allow researchers to build more easily upon the work of others.

### 4.3 Playing FAIR

The FAIR (Findable, Accessible, Interoperable, Reusable) principles provide a framework to optimize the use of existing data research. These principles maintain that datasets must have a persistent identifier (for example, a Digital Object Identifier, DOI) and sufficient metadata (such as information about the data, including the date, methods, standards, and accuracy) to make them Findable. Data should be archived in data repositories and have procedures in place to request access, making them accessible. To increase interoperability, data should be stored in open formats (to increase longevity of the data) and follow a common pattern and consistent vocabulary, including those used for metadata, making them understandable to both machines and humans. Finally, data should be accompanied by a license and documentation of their provenance (which in the case of isotopes may include laboratory notes and protocols), making the dataset reusable (Plomp et al., 2022; Wilkinson et al., 2016). Here, we do not dwell on the particulars of making isotopic data interoperable and reusable as currently many publications detail up‐to‐date guidelines for reporting stable isotopic analyses, including several specific to archaeology (Roberts et al., 2018; Szpak et al., 2017; Vaiglova et al., 2022). Instead, we focus on how to make isotopic data findable and accessible, namely via intentional and conscientious sharing.

### 4.4 Ensuring access for all

Besides sharing data through online repositories, data management should consider how to make said data accessible to other rights holders outside the traditional scientific community, including research participants, descendant communities, policy‐making institutions, and society at large. For instance, by sharing data copies and short reports with any partnering institutions, museums, laboratories, or individual researchers. These partners should have a working idea of what these datasets contain and how they are organized so that they may be able to navigate new requests for sample access (Vaiglova et al., 2022). Additionally, researchers should cultivate long‐term relationships with their partners so that they are available to clarify any doubts that may arise further down the line; there are many ways these relationships may take shape in relation to data management and sharing but could include agreeing on a schedule of regular updates on data acquisition and initial findings, as well as regular reminders what channels of communication are always available. When working with partners at different points of the knowledge‐power gradient (McKerracher & Núñez‐de la Mora, 2022), this may often translate into contributing to the development of local databases, either financially or through labor. Particular care should be taken when research involves communities often excluded, as both producers and consumers, from the mainstream of knowledge. Here, the CARE (Collective Benefit, Authority to Control, Responsibility, and Ethics) principles provide a framework to manage the data appropriately (Carroll et al., 2020). In line with these principles, we encourage researchers to explore the possibility of securing a Label or Notice hosted through the Local Contexts Hub, an initiative funded in 2010 that seeks to increase Indigenous involvement in data governance (About–Local Contexts, n.d.). The labels and notices are “a highly visible, machine‐readable, persistent and durable connection between collaborating Indigenous communities, and researchers, research projects and activity” (Liggins et al., 2021, p. 2479) and signal the provenance of a dataset from a descendant community and the rights of these communities to define the future use of data and derived benefits. At the moment, this initiative centers around genetic data from Indigenous communities residing in English‐speaking countries, but we believe it would be of key interest to all involved to expand its application to other contexts and types of datasets.

### 4.5 Sharing is caring

Nowadays there are many ways to share data online (Table 1). Both IsoBank and IsoArcH are responses to the increased output in isotopic publications in the last few decades (Pauli et al., 2017; Roberts et al., 2018) and both offer some guidelines on how to share isotopic data in interoperable ways. Furthermore, the IsoArcH project team openly subscribes to the FAIR and CARE principles (Plomp et al., 2022). Besides IsoArcH and IsoBank, there are use‐specific databases with tailored variables that hold strength for targeted research but may lack long‐term support management compared to larger inter‐institutional repositories, leading to orphaned data. Compared to DNA's international public repositories (GenBank in the United States, DNA Databank of Japan, and the European Nucleotide Archive) with governmental support and routine data‐sharing across platforms (*GenBank Overview*, n.d.), isotope archaeology has far to go. It is also possible to use other general open repositories such as Zenodo (*Zenodo–Research. Shared*. 2023), Figshare (*Figshare—Credit for All Your Research*, 2022), and OpenContext, a repository focusing on archeological data (*Open Context*, 2022). There are also community led projects, such as Mukurtu, which seeks “to empower communities to manage, share, narrate, and exchange their digital heritage in culturally relevant and ethically‐minded ways” (About–Mukurtu CMS, n.d.). These smaller data storage systems, such as Mukurtu, have the same potential issues as mentioned for use‐specific databases but may have more refined systems for controlling data access. For some data, this issue supersedes FAIR principles.

**TABLE 1:** Examples of current data repositories and databases focusing on the collection of isotopic data.

Name	Description and focus	Reference
IsoArcH	Data repository focusing on archeologically‐derived isotopic data, with an associated nonprofit academic association	(Plomp et al., 2022; Salesse et al., 2018)
IsoBank	Data repository gathering isotopic data from any context (ecological, experimental, archeological)	(Pauli et al., 2015)
Bitacora	Database with modern human teeth and keratin isotope values	(Valenzuela et al., 2023)
Archipelago	Database of carbon and nitrogen stable isotope values of humans from Japan	(Fernandes et al., 2021)
Amalthea	Collection of human tooth increment isotopic data	(Cocozza & Fernandes, 2021)

### 4.6 When not to share

In line with this, we recognize that there are situations when it is not ethical or appropriate to share data. Some reasons to not share the data openly would be: if the community the data belongs to does not want the data to be shared (see considerations in Mengoni Goñalons and Figuerero Torres 2023), if the data could lead to the identification of an individual or living descendant(s), or if the remains have been obtained in unethical ways (Alves‐Cardoso & Campanacho, 2022). A mid‐way solution could be to implement additional policies to ensure the proper use of the data, via access management by individuals and communities involved. For example, if only the information about the data (metadata) is shared publicly, but individuals would have to follow a procedure to get access, the data would still follow the FAIR principles. We also acknowledge that descendant or rights holder communities are not monolithic and that different views on data sharing may arise both between and within communities; the particulars will differ on a case‐by‐case basis, requiring researchers to build relationships of trust and ensure continued open communication. Once again, these conversations take time, further strengthening our call for “slow science.”

What to do when a dataset is flagged for ethical issues? Or when informed consent is withdrawn? How does a project build in governance around rights of refusal? Considering that informed consent is an ongoing dialogue and not a pinpoint in time (Turner et al., 2018), researchers and data management plans should be amenable to withdrawing their dataset from open repositories. Besides this personal responsibility, it is difficult to ascertain how institutions, funding bodies, and data repositories should navigate such issues. A general rule of thumb would be to remove datasets from public access until a more thorough review process is possible should the complaint yield from a less privileged party along the knowledge‐power gradient. Obviously, this generalist guideline immediately creates follow‐up issues. Identities are intersectional and position along the knowledge‐power gradient is always relational and often not clearcut. In addition, it will be difficult for any organization to arbitrate between parties to achieve the aforementioned thorough review, especially those without independent budgets, as is the case with many open access data repositories. Ethics‐centered working groups have developed recommendations toward best practices to handle ethical cases relating to the sharing and publication of research data (Puebla et al., 2021).

## 5 THE SCIENTIFIC ECOSYSTEM

**FIGURE 2:**
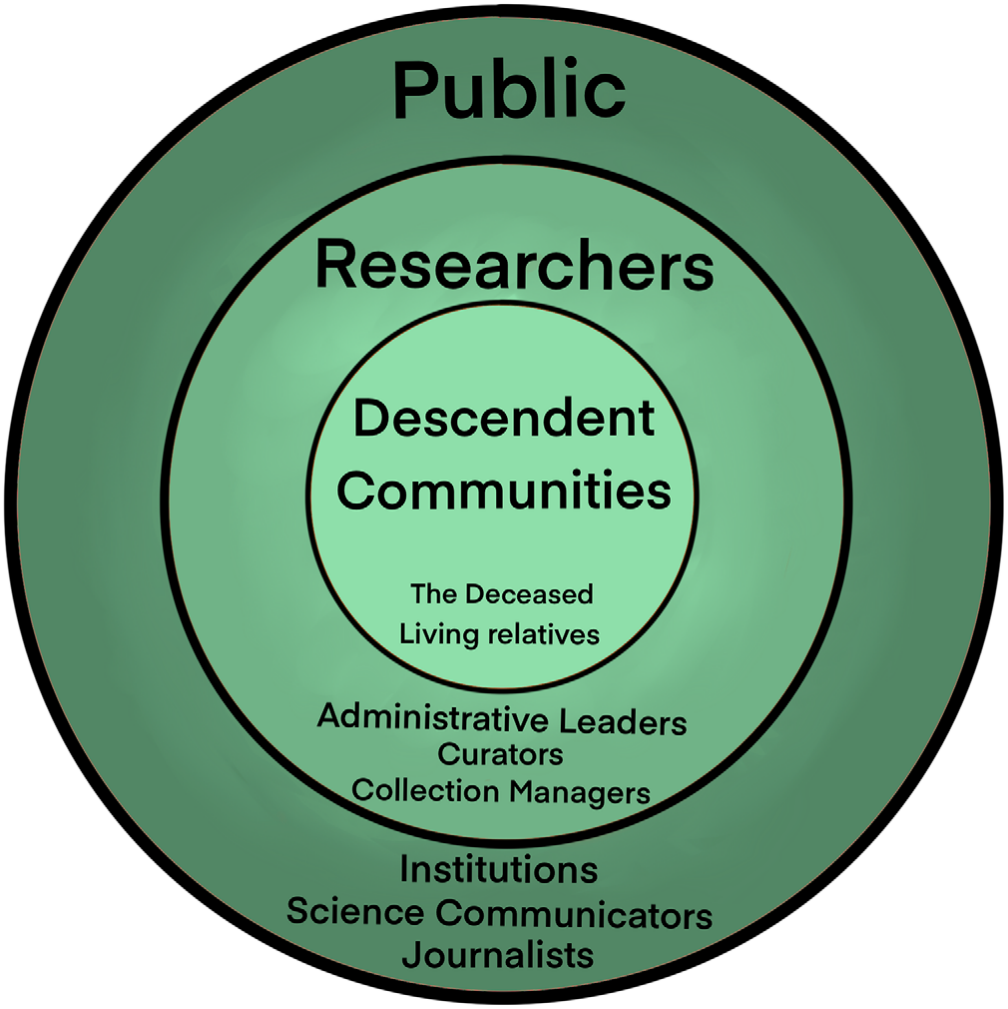
Inspired by Gayle Rubin's “Charmed Circle” (2002) and Sylvia Duckworth's wheel of power/privilege (n.d.), we visualize the active players in ethical research, with intentional centering of those who should be prioritized. The shades are intentionally graduated to highlight that there are people within multiple bands of (for example, science communicators who are also living relatives).

We intentionally begin and end with communities, creating a cyclical model of ethical research (Figure 2). Descendant communities and other ethical rights holders, academics, and broader groups engaged through social media and news all create an ecosystem of interested parties who shape both the research's design and legacy. For example, a multidisciplinary investigation, which included stable isotope analysis of a Coast Salish dog began when the principal investigator was inspired by a magazine article to help combat colonial narratives pervading scholarly writing on the decline of this dog breed (Bidal, 2023; Lin et al., 2023). To this end, we finish our paper thinking about the ways in which isotope researchers can contribute to creating an open dialogue with other interested groups.

### 5.1 Isotopes in the public eye

It is every researcher's goal to produce research with real impact. However, while isotope research can offer valuable insights and clarity to questions of human behavior, it is also open to being misinterpreted or misconstrued by external parties, such as journalists or private individuals in ways that can cause real harm. In the most benign of these scenarios, isotopic data may be overinterpreted (often in the media) to claim more certainty than can be offered by methodological limitations, resulting in “hardening of the story whereby the nuances of interpretation are lost in the need to create good stories” (Pollard, 2011, p. 631). At the more extreme end of the spectrum, anthropological and archeological evidence for chronology, human mobility, and even diet may be used in bad faith to promote the politicized narratives of partisan media or resurgent ethnonationalist groups (Gambert & Linné, 2018). These issues have been noted as frequent hazards for paleogenetic research, where genetic analyses may be used in support of essentialist conflation of genomic links and ethnic identity (Hakenbeck, 2019).

These same risks are present in isotopic research. Work tracing the movement of people and pigs during the Neolithic near Stonehenge in the UK (Madgwick et al., 2019) was grabbed by news outlets and interpreted through the lens of Brexit, the withdrawal of the United Kingdom from the European Union. Important responses prepared by the original researchers decried this comparison and its misguided use (Madgwick et al., 2021), leading to a back‐and‐forth between journalists and scholars on the nature of analogy and how it is used for archeological case studies. As archeologists, anthropologists, and broadly interdisciplinary scholars, we seek to connect our work to current events in order to relate our narratives to the present and future. In the case of the Brexit comparison to evidence of migration in Neolithic Britain, we can learn that even flippantly, analogies can take on lives of their own.

The case of Madgwick and colleagues in the 2019 original article and 2021 rebuttal to the press and others could be seen as an example of why learning to communicate to specialists and nonspecialists, in addition to having promotional utility, is an ethical obligation. While the Brexit comparisons to the Neolithic had ultimately little impact on the existing public perception of British isolationism and exceptionalism, it highlighted some key considerations in engaging with other communities/groups, both the media and the general public. Madgwick et al.'s work was reported to the media through press releases along with public and academic talks, so information that is reiterated in different ways to different audiences can be co‐opted for different reasons, many of them motivated by political movements. Nevertheless, the research team was admirably quick and clear in their response when blindsided by unexpected narratives. This well‐known example of archeological isotopic research being employed within the context of a heated political debate highlights the need for better‐entrenched training in media engagement and writing clearly to prevent the repetition of these instances in the future. In order to encourage scientific communication and literacy outside academic spheres and increase scientific comprehension, we suggest that the teaching of science communication, or organizing its teaching by experts, be integrated into both undergraduate and graduate‐level training.

Beyond publications and traditional journalism, it is increasingly common for researchers to curate a multi‐media digital presence; websites, social media, YouTube, and the like, to both share their own research and participate in scientific communication more broadly (Huffer, 2018; Killgrove, 2019; Stojanowski & Duncan, 2015). As we broadcast our work through traditional publications and emerging science communication platforms, isotopic expertise can also attract attention from collectors and dealers within the global antiquities and human remains trade who might seek not only general osteological or paleopathological information about remains currently in their possession, but also deliberately contact individuals affiliated with laboratories to request that “samples” they remove (usually incorrectly) undergo specific analyses. One way that isotope specialists can contribute to combatting the mishandling or illegal export/import of human remains is for isotope geochemistry laboratories to always do due diligence, requiring anyone who approaches them for small contracts first provide thorough documentation demonstrating that they have obtained the necessary permissions and are submitting samples on behalf of a public institution. This is especially important if the individual wishing to submit samples claims to be acting on behalf of a consignor to an auction house, which should raise a red flag to begin with. Previous and ongoing research has established that today's human remains trade rests on the same expressly colonial, extractionary, foundations as bioanthropology itself (Redman, 2016). Indeed, collector attitudes and practices today, on or off‐line, often draw heavily on Victorian‐era rhetoric or aesthetics when remains or personal “Wunderkammers” are shown off or advertised across numerous platforms (Davidson et al., 2021; Huffer & Chappell, 2014).

While most bioarcheologists or forensic anthropologists affiliated with laboratories for purposes of isotopic research might never personally receive a request from a collector, a fully informed ethical isotopic bioarcheology must consider this possibility. As open access publishing increasingly shows the world the capabilities and limitations of isotopes, in terms of understanding provenience and life history, the potential for laboratories being approached by collectors seeking more context to fetch higher prices might increase. An ethical isotopic bioarcheology must ensure that all involved actively mitigate the risk of inadvertently “authenticating the market,” thereby allowing the seller to profit from the sale of human remains (or antiquities or Indigenous cultural heritage often referred to as “tribal art”), frequently of illicit or unethical origins.

### 5.2 Sustaining academic community

We urge researchers to consider how they engage with each other; the scientific ecosystem must provide a safe and supportive environment for all, especially early career researchers, researchers from the Global South, and those with marginalized social identities. As connected members of interdependent networks, researchers have obligations to each other that equally promote good collegial relationships and good science. Isotope research, with its specialized analytical knowledge and finicky instrumentation, increasingly requires teams of people to provide their contributions across chemical, osteological, and cultural spheres of knowledge. These relationships can create great scholarship but can allow unequal power relationships that enable harm.

The creation of a safe and supportive environment starts with students. There are cases in higher education where under‐represented or racialized minority communities often first enter university through less‐prestigious institutions that often lack the means to fund important research programs through field schools and academic work (Fry & Cilluffo, 2019). American anthropology departments have been identified as reinforcing systematic inequality that maintains these barriers (Kawa et al., 2019). In these cases, funding opportunities may be limited to organizations with an international remit, such as the Wenner‐Gren Foundation, Ford Foundation, and National Geographic. However, access to such funding is not direct and requires extensive knowledge of academic English, which ends up posing challenges for the inclusion of students from less privileged social groups and/or second‐language English speakers (Bortolus, 2012).

One of the discipline‐wide issues contributing to environments unconducive to good scholarship practice is the pressure to create as many peer‐reviewed publications as possible. Sometimes called a “push to publish,” “publish or perish,” or a “paper mill,” this pressure for high output is felt throughout archeology but especially pushed in archeological sciences (Pilaar Birch & Szpak, 2022). Rooted in destructive and unequal capitalist practices, and paired with the increasing insecurity of contingent labor in academia, it is easy to find ourselves without: without time to think, without time to weigh options, and without time to make informed ethical decisions (Cunningham & MacEachern, 2016).

Dismantling the hegemony of neoliberalism in academia is a daunting task. However, many of the ways to move toward an inclusive community are not onerous tasks but rather labors of joy. Uplift and celebrate those doing good work, especially those whose work is so often pushed to the margins of scholarship. Peer‐review papers and grant applications with the intent to improve the research, not to belittle the researchers. Positive outcomes in mentorship are key to enabling autonomy and creative problem‐solving in archaeology (Brown, 2018). Those with the ability to do so must expand their circle of scholarship to include those not traditionally included in, or actively excluded from, publication: student technical assistants, descendant community representatives, and others who can provide multidisciplinary and blended theoretical perspectives. Program officers, grant reviewers, and others in this sector have the ability to make major culture shifts in the discipline if these types of activities become priorities.

## 6 CONCLUDING THOUGHTS

This paper arose from the question: what are the levels of care and respect we should integrate into our research design and practice as isotopes specialists? During the creation of this piece, as collaborators joined and contributed their own experiences, we saw the multi‐faceted situations we encountered across the world. Can we get the discipline to agree on an approach when even within this collaborative group there may be differing views? This collaboration started with a focus on the ethical considerations surrounding the isotopic analysis of human remains from archeological contexts. Most of the co‐authors would identify that as the focus of their research. But almost every collaborator on this paper has encountered ethical considerations outside of the main focus, which suggests that our readers possibly will too. And so, we touched on issues relating to heritage crime, forensic contexts, and modern samples.

Additionally, we must acknowledge that many of the “ideal” recommendations made here are not without problematic assumptions of sufficient researcher experience and equitable access to resources and power. For instance, many of the pre‐screening methods listed are time and resource expensive, and in practice often only used when studying extremely rare and valued materials, such as hominin fossils. Enforcing them as mandatory could, among other unintended repercussions, aggravate international inequalities, wherein researchers from countries with fewer research resources could be further excluded from the production of scientific discourse. In instances where ethical concerns are raised that require pausing research or stopping questionable practices altogether, junior researchers, who often produce the bulk of data, may jeopardize their personal and professional well‐being when standing up against academic power structures. Our goal with this article has been to strengthen the position of researchers and communities that value ethical approaches in science but we do so without pretending that the world is not complex.

The field of isotopes in archaeology has now been around for more than four decades, and we hope this work provides a snapshot of the current zeitgeist of the discipline: considerations of ethical situations routinely faced today and proposals for actions that can create a more equitable future.

## AUTHOR CONTRIBUTIONS

**Chris Stantis:** Conceptualization (lead); project administration (lead); writing – original draft (equal); writing – review and editing (equal). **Ben Schaefer:** Visualization (lead); writing – original draft (equal); writing – review and editing (equal). **Maria Ana Correia:** Visualization (equal); writing – original draft (equal); writing – review and editing (equal). **Aleksa K. Alaica:** Writing – original draft (equal); writing – review and editing (equal). **Damien Huffer:** Writing – original draft (equal); writing – review and editing (equal). **Esther Plomp:** Writing – original draft (equal); writing – review and editing (equal). **Marina Di Giusto:** Writing – original draft (equal); writing – review and editing (equal). **Blessing Chidimuro:** Writing – original draft (equal); writing – review and editing (equal). **Alice K. Rose:** Writing – original draft (equal); writing – review and editing (equal). **Ayushi Nayak:** Writing – original draft (supporting); writing – review and editing (supporting). **Ellen Kendall:** Conceptualization (equal); writing – original draft (equal); writing – review and editing (equal).

## FUNDING INFORMATION

MAC was supported by the Portuguese Foundation for Science and Technology (DOI: 2022.03020.CEECIND/CP1731/CT0006).

## Data Availability

No new data was generated for the creation of this manuscript.
